# A Case of Leptomeningeal Carcinomatosis Manifesting With Vertigo in the Setting of Intracranial Hypertension

**DOI:** 10.7759/cureus.47431

**Published:** 2023-10-21

**Authors:** Leena Saeed, Sondos K Khalil, Sulafa K Khalil, Omar A Madani, Fakhreddin Al Refai, Muzamil Musa, Ehab Adam, Husam Abu-obieda

**Affiliations:** 1 Internal Medicine, Hamad Medical Corporation, Doha, QAT; 2 Medicine, University of Gezira, Khartoum, SDN; 3 Neurology, Hamad Medical Corporation, Doha, QAT

**Keywords:** leptomeningeal carcinomatosis, carcinomatous meningitis, leptomeningeal disease, vertigo, intracranial hypertension, leptomeningeal carcinomatosis (lmc)

## Abstract

Leptomeningeal carcinomatosis (LMC) is a rare condition where malignant cells infiltrate the leptomeninges of the central nervous system. We present a case of a 51-year-old male with stage IV adenocarcinoma of the lung who developed recurrent vertigo. The patient initially received a diagnosis of peripheral vertigo, but his symptoms worsened over time and were associated with headache, vomiting, and one episode of seizure. Upon readmission, based on his normal neuroimaging results, normal CSF examination with elevated opening pressure, and papilledema on fundoscopic examination, a diagnosis of pseudotumor cerebri was made. The result of CSF cytology revealed the presence of malignant cells confirming the presence of LMC. This case highlights the importance of considering LMC as a potential cause for unusual neurological symptoms in patients with advanced malignancy, particularly when other conditions like pseudotumor cerebri could obscure its presentation. It is crucial to rule out malignancy through CSF cytology in patients presenting with vertigo and/or other vestibulocochlear symptoms before making an alternative diagnosis that could present similarly.

## Introduction

Leptomeningeal carcinomatosis (LMC) (also known as leptomeningeal disease/carcinomatous meningitis) is a disease in which malignant cells infiltrate the leptomeninges of the CNS. Leptomeninges refer to the part of the meninges that includes the arachnoid membrane and the pia mater, while the dura matter comprises the pachymeninges [1]. Malignant involvement of the pachymeninges is referred to as pachymeningeal carcinomatosis.

Multiple mechanisms by which malignant cells gain access to the CSF have been described. Direct spread from brain parenchyma and hematogenous dissemination via arachnoid veins are the most common. Other mechanisms include metastasis from the choroid plexus, perineural and endoneurial invasion, and cerebellar extension following neurosurgical resection. Overexpression of C3 protein, which in turn enhances endothelial proliferation and thus provokes metastasis, has also been described [2]. There are different patterns of leptomeningeal involvement. The most involved regions are basal cisterns, the posterior fossa, and the cauda equina. This is explained by the slow CSF flow and gravitational effect in these areas aiding in the disposition of migrating cells [1,2].

LMC presents a wide range of clinical manifestations and can affect both CNS and peripheral nervous system (PNS) [3]. Symptoms and signs of LMC are due to the involvement of the brain hemisphere (15%), cranial nerves (35%), and spinal cord and nerve root (60%) [4,5]. Signs of intracranial hypertension are also common (26%) [6].

We are reporting a rare case of LMC of a 51-year-old male with a known history of advanced lung cancer presenting with vertigo in which initial investigations favored pseudotumor cerebri. However, later CSF cytology confirmed LMC.

## Case presentation

We present a case of a 51-year-old gentleman, who was a known case of stage IV adenocarcinoma of the lung. He was first diagnosed with lung cancer in 2017 and received chemotherapy and radiotherapy to the left upper lobe and mediastinum. Following these treatments, he has been receiving bevacizumab as maintenance therapy. He presented to the emergency department with a chief complaint of vertigo. Vertigo episodes were primarily triggered by head movements on both sides and were accompanied by recurrent episodes of non-bilious non-projectile vomiting and headache that started in the occipital region and spread to involve the entire head. There was no weakness or numbness, no visual disturbance, no urinary or fecal retention or incontinence, no changes in mood, behavior, or memory, no other neurological symptoms, and no fever, chills, or a history of head trauma. The systemic review was significant for one episode of tinnitus in the left ear that started on the same day of presentation, with no changes in hearing or ear discharge.

The patient described a history of one episode of severe headache associated with jerky movement affecting all limbs, resulting in a loss of responsiveness. This happened at home and was witnessed by his son. The patient had been admitted one week prior with similar complaints of vertigo, headache, and vomiting and was diagnosed with acute right thalamic infarction, for which he was started on aspirin and statin, and was referred to neurology and ENT for evaluation of vertigo. Assessment by neurology and ENT three days prior to admission concluded a probable diagnosis of peripheral vertigo.

On clinical evaluation, the patient was afebrile (36.8°C), normotensive (128/86 mmHg), with a pulse rate of 89 beats/minute, respiratory rate of 18 breaths/minute, and maintained oxygen saturation on room air. Neurological examination revealed an alert patient, oriented to time, place, and person, with a Glasgow Coma Scale (GCS) score of 15/15, and a higher mental examination was normal. Cranial nerves were intact, and no nuchal rigidity was appreciated. He had normal muscle bulk with no apparent or elicited fasciculations, skin changes, or deformities, normal tone, and power bilaterally in upper and lower limbs with normal tendon reflexes, absent Babinski sign, and no ankle clonus bilaterally. Sensations were normal, with normal coordination. Assessing the gait was difficult because of the vertigo. Chest, precordium, and abdominal examinations were all normal. Initial laboratory tests are shown below (Table 1).

**Table 1:** Initial blood tests. WBC: white blood cells; Hgb: hemoglobin; INR: international normalized ratio; APTT: activated partial thromboplastin time; ALT: alanine transaminase; AST: aspartate transferase.

Test	Result	References
WBC	5.3	4.0-10.0 x10^3^/uL
Hgb	15	13.0-17.0 gm/dL
Platelet	175	150-400 x10^3^/uL
Prothrombin time	11.9	9.7-11.8 seconds
INR	1.1	>4.9 critical high
APTT	25.4	24.6-31.2 seconds
Creatinine	92	62-106 umol/L
Sodium	134	136-145 mmol/L
Potassium	4	3.5-5.1 mmol/L
Adjusted calcium	2.41	2.15-2.50 mmol/L
Bilirubin total	7	0-21 umol/L
Albumin	37	35-52 gm/L
Alkaline phosphatase	83	40-129 U/L
ALT	18	0-41 U/L
AST	Hemolyzed	0-40 U/L
Lipase	26	13-60 U/L
Lactic acid	2	0.5-2.2 mmol/L
Blood glucose	6.7	3.3-5.5 mmol/L

**Figure 1:**
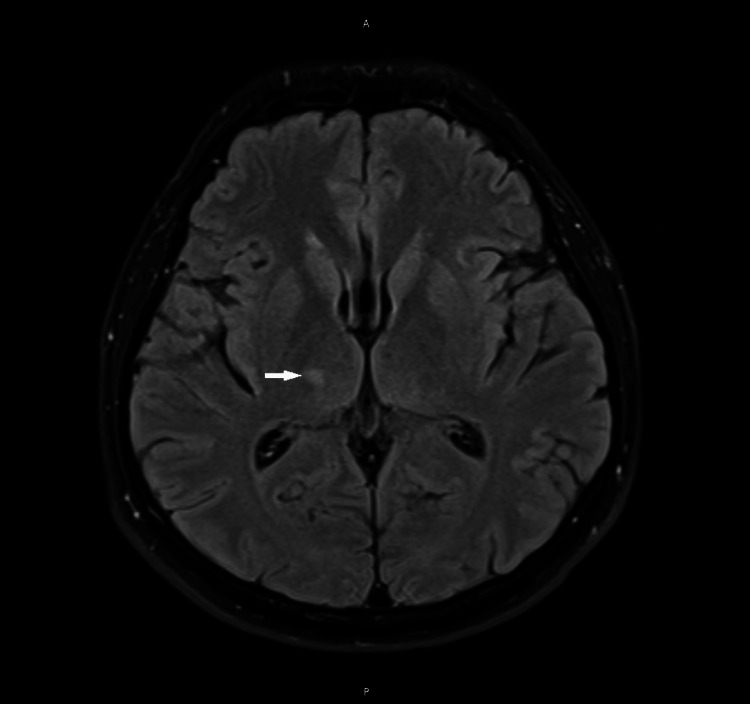
Partially restricted diffusion with diffusion-weighted imaging at the right thalamic area (white arrow).

MRI of the brain was not done because it was performed one week before admission to the ED for the same complaints and showed a right thalamic infarct (Figure 1). However, this does not explain his persistent vertigo. No other pathology was seen at that time. Magnetic resonance cerebral venography was done to exclude an early hypercoagulable state in a cancer patient but no evidence of cerebral vein thrombosis was found.

During hospital admission, his vertigo and headache worsened, and he began to have high readings of blood pressure. On the fifth day of admission, he experienced an episode of jerky movement in the right hand and a lack of awareness that lasted for around 30 seconds. The physician on-call examined the patient in the post-ictal phase. He had slow speech but was oriented and stable. The son showed a video of his father to the doctor, which showed vacant staring episodes associated with nystagmus. He also complained of blurring of vision.

Fundoscopic examination, EEG, and lumbar puncture were done. Fundoscopic examination revealed bilateral grade 3 optic disc swelling, peri-papillary hemorrhage, and cotton wool spots. On lumbar puncture, the opening pressure was 53 cm H2o, and the sample was sent for analysis and cytology (Table 2); however, therapeutic CSF removal was not done. EEG showed a well-formed symmetrical alpha rhythm at 8-9 Hz with electrographic features of mild drowsiness. No focal abnormalities or epileptic discharges were observed, and photic stimulation showed normal responses.

**Table 2:** Initial CSF analysis report. CSF: cerebrospinal fluid; RBC: red blood cells; TB: tuberculosis; PCR: polymerase chain reaction; HSV: herpes simplex virus; N/A: not applicable.

Test	Result	References
CSF color	Colorless	N/A
CSF appearance	Clear	N/A
CSF total nucleated cell	2	0-5 uL
CSF RBC	Nil	0-2/uL
CSF glucose	3.50	2.22-3.89 mmol/L
CSF protein	0.18	0.15-0.45 gm/L
CSF albumin	64	0-350 mg/L
CSF culture	No growth	N/A
CSF TB PCR	Negative	N/A
CSF HSV 1 and 2, varicella zoster, mumps, parechovirus, enterovirus PCR	Negative	N/A
CSF cryptococcal antigen	Negative	N/A
CSF paraneoplastic autoantibody evaluation	No autoantibodies were detected	N/A

The patient’s symptoms improved significantly after the lumber puncture, as he reported dramatic improvement in headache, and did not experience abnormal body or eye movements again. Given the high opening pressure and the improvement of symptoms following lumbar puncture, a preliminary diagnosis of pseudotumor cerebri was made, although the patient does not have any risk factors for such a diagnosis. The patient was started on acetazolamide 500 mg three times a day (TID) and kept for observation. After two days of being symptoms-free, and four days following lumbar puncture, the patient was discharged on acetazolamide 500 mg TID and Keppra 500 mg twice a day (BID). The patient was booked for follow-up with the neurology/epilepsy clinic to follow CFS cytology results and seizure.

Three days after discharge, the CSF cytology was available. It revealed the presence of highly atypical cells that were suspicious of malignancy.

The patient presented again to the ED eight days after discharge with severe nausea, vomiting, and dizziness. His symptoms were not responding to treatment given on discharge. The patient also had high troponin T and ECG changes and was assessed by the cardiology team, who determined that the patient was experiencing type II myocardial infarction. The patient was placed on dual antiplatelet therapy and statins.

After stabilization, the patient was transferred to the oncology team to start intrathecal chemotherapy for LMC and to ensure continuity of care. He received his first cycle of intrathecal methotrexate inpatient and was discharged after a total admission of 13 days. Despite receiving weekly chemotherapy, the patient's condition continued to deteriorate, leading to the development of quadriplegia. Consequently, chemotherapy was discontinued. Three months later, he was readmitted with headache and seizure. Unfortunately, he passed away shortly after admission.

## Discussion

LMC occurs in 5-8% of patients with systematic malignancy [7,8]. Breast cancer has been considered the leading cause of LMC accounting for 11-64% of the overall incidence of LMC in solid tumors, followed by lung cancer (14-29%) and melanoma (6-18%) [9-12]. These numbers are highly influenced by the overall incidence of these malignancies in the world population [1].

LMC presents with a wide range of neurological symptoms. A study conducted by Jayson et al. found that headaches, lower limb weakness, cerebral symptoms, and involvement of the cranial (with CN III, IV, VI, and VII) and spinal nerves were the commonest presentations; other presentations include meningism, cerebellar signs, nausea/vomiting, altered mental state, and autonomic dysfunction. Normal examination has also been described [13].

LMC diagnosis can be made based on neuroimaging and CSF examination. Gadolinium-enhanced MRI of the brain and spine may show linear or nodular enhancement of affected areas. MRI is more sensitive than contrast-enhanced CT scan, making it the modality of choice; if there are any contraindications to MRI, CT can be done. MRI must be done before lumbar puncture or any shunt procedure as they can cause meningeal enhancement leading to false positive results [14]. CSF examination will show the classical findings of elevated protein concentration, decreased glucose, leukocytosis with lymphocytosis, and a CSF cytology positive for malignant cells. Of these findings, only glucose reduction is seen in malignancy and infections involving leptomeninges, the rest of the findings are considered non-specific [1,13]. In our patient, none of these findings could be seen, as his MRI one week before presentation showed a thalamic stroke. No other pathologies were seen, and CSF analysis was within lab references.

CSF cytology is a specific test for LMC, and around 50% of patients will have positive cytology on the initial lumbar puncture; this number can rise to 85% in three high-volume lumbar punctures [13]. On the other hand, a normal CSF profile could be seen in less than 5% of the patients, making it a good negative predictor for the disease [15]. Our patient's CSF cytology reported positive on the first lumbar puncture.

Vertigo is a common symptom that can co-exist with multiple conditions; however, it is a rare manifestation of malignancies of the CNS, including brain tumors and LMC. It is more related to pathologies in the inner ear. Currently, there are no data available on the incidence of vertigo in LMC. However, based on our review of the literature in PubMed, we identified three individual case reports, and one case series with two cases reporting patients with a history of malignancy presenting with vertigo, who were found to have leptomeningeal disease. Vitaliani et al. reported a case of a 63-year-old female diagnosed with ovarian cancer who presented with subacute right deafness, vertigo, and imbalance, though, unlike our patient who had no findings suggestive of LMC on MRI, enhancement of the CN VIII could be seen in the MRI of the patient [16]. Jarabin et al. reported a 51-year-old male with a history of gastric carcinoma treated with partial gastrectomy who presented with vertigo, headaches, nausea, and vomiting. His MRI showed enhancement of the left petrous bone but was otherwise normal. CSF examination showed elevated total proteins, hypoglycemia, and pleocytosis. Cytology was negative for malignancy, in contrast to our patient who had normal CSF analysis, but cytology was positive for malignant cells. After a complicated course, the patient passed away, and the autopsy confirmed infiltration of meninges by malignant cells [17].

The mechanism by which LMC can cause vertigo is still not fully understood. Vitaliani et al. suggested that CN VIII or cerebellar involvement could explain vertigo, and in their case, this was proven by cranial nerve VIII nodular enhancement in the internal acoustic meatus and pial enhancement in the cerebellar sulci [16].

Another possible mechanism is elevated intracranial pressure (ICP) altering perilymphatic pressure and ultimately endolymphatic pressure, due to communication between these spaces; any change in CSF pressure such as raised ICP will be mirrored in the inner ear pressure and manifest with vertigo and other cochleovestibular associated symptoms [18]. This mechanism could possibly explain the presentation in our patient, given the fact that there were no changes observed in his neuroimaging studies that could explain his symptoms. Elevated ICP due to LMC can manifest in multiple ways; pseudotumor cerebri [19-21] and intracranial hypertension with hydrocephalus [22] have been reported in the literature.

This case report is a powerful reminder of the necessity to consider underlying LMC as a potential etiology for new-onset neurological symptoms in cases where advanced primary cancer is already established. LMC rarely presents vertigo. Thus, it is unfortunately plausible for such cases to be missed, especially when an alternate diagnosis that could present similarly is evident in the clinical context, thus maintaining a heightened clinical suspicion remains paramount to prevent delayed diagnosis of such cases.

## Conclusions

Our patient received the diagnosis of peripheral vertigo after visiting the ED multiple times. Upon admission, investigations that included head imaging and CSF analysis concluded intracranial hypertension, and a diagnosis of idiopathic intracranial hypertension was made, which was questionable. Subsequent cytology results available after the patient's discharge disclosed the presence of leptomeningeal malignancy. We recommend investigating LMC patients with a history of malignancy presenting with vertigo before attributing the presentation to an alternative diagnosis that may mimic the disease presentation to reduce morbidity. We also like to draw physicians' attention to the possibility of normal CSF analysis findings in patients with LMC.
